# Valorization of Phosphorus Secondary Raw Materials by *Acidithiobacillus ferrooxidans*

**DOI:** 10.3390/molecules22030473

**Published:** 2017-03-16

**Authors:** Małgorzata Wyciszkiewicz, Agnieszka Saeid, Przemysław Malinowski, Katarzyna Chojnacka

**Affiliations:** 1 Department of Advanced Material Technologies, Faculty of Chemistry, Wroclaw University of Technology, Smoluchowskiego 25, 50-372 Wroclaw, Poland; malgorzata.wyciszkiewicz@pwr.edu.pl (M.W.); katarzyna.chojnacka@pwr.edu.pl (K.C.); 2 Basic Science Center, University of Applied Sciences in Nysa, Armii Krajowej 7, 48-300 Nysa, Poland; przemyslaw.malinowski@pwsz.nysa.pl

**Keywords:** biosolubilization, fish bones, phosphorite, phosphate biofertilizers, poultry bones

## Abstract

This paper presents the possibility of producing phosphorus fertilizers through *Acidithiobacillus ferrooxidans* utilization in secondary raw materials solubilization. Phosphorus was obtained from the bones of poultry and fish as well as from Morocco phosphorite. Four doses of poultry bones and fish bones were used in the experiment (2, 4, 10 and 20 g/L) and two doses (2 and 4 g/L) of phosphorite were also used. The experimenters measured the final pH, which increased in proportion to the increase in the number of poultry bone doses, whereas in the case of fish bones it decreased in proportion to the increase in the number of fish bone doses. Only in the case of phosphorite, where 10 g/L were used, there was a slight increase in pH during solubilization observed. The highest phosphorus concentration of 1.9% (expressed as P_2_O_5_) was found for the solubilization performed on fish bones with the highest dose (20 g/L). The formulation obtained in this study meets the necessary requirements for use as a bio-fertilizer because of the relatively low content of P_2_O_5_ and the low content of toxic elements. The results confirm the utilization of *Acidithiobacillus ferrooxidans* in the biosolubilization of phosphorus renewable raw materials that can alleviate the problem of the world’s depleting phosphorite deposits.

## 1. Introduction

The manufacturing costs of mineral fertilizers largely depend on the price of raw materials, which could be reduced if renewable by-products could be used [1]. Limited resources of natural phosphates and the unstable geopolitical situations in the regions where the largest deposits of phosphorites are located have made the recovery of phosphorus from wastes rich in fertilizers of utmost importance in recent years [2].

A wider application of biotechnologies to activate phosphate raw materials may help reduce the adverse phenomena attendant on the manufacturing of conventional fertilizers [3]. These processes can involve bacteria and fungi that solubilize phosphate (Phosphate Solubilizing Bacteria—PSB, Phosphate Solubilizing Fungi—PSF) [4].

PSB and PSF are integral parts of the phosphorus cycle in soil [5]. PSB are chiefly responsible for phosphorus release from organic and inorganic pools by solubilization and mineralization and are more efficient than PSF [6]. *Pseudomonas*, *Bacillus*, *Rhizobium*, *Burkholderia*, *Achromobacter*, *Agrobacterium*, *Microccocus*, *Aerobacter*, *Flavobacterium* and *Erwinia* are among the bacteria strains that change such forms of inorganic phosphorus as tricalcium phosphate, dicalcium phosphate, hydroxyl apatite and rock phosphate into forms available to plants [4]. PSB activity is driven by mechanisms producing organic (such as citric, gluconic, lactic, and propionic) as well as inorganic (such as sulfuric) acids [7]. *Thiobacillus ferrooxidans* and *Acidithiobacillus thiooxidans* raise the greatest expectations because they produce sulfuric acid [8,9].

In order to make phosphorus from soil available for plants, the organic compounds must be mineralized, which in most cases requires enzymes such as phosphatase (phosphohydrolases), phytase, phosphonoacetate hydrolase, and d-α-glycerophosphatase [4].

The use of microorganisms for phosphorus solubilization was first patented in 1910 [10]. It proposed the utilization of bacteria from disintegrated, weathered rock for activating phosphorus from phosphate. Fertilizers containing PSB can be manufactured in liquid and solid form. The former contains phosphorus compounds activated by bacteria at the manufacturing stage. In this type of fertilizer, phosphorus can be obtained from different, mostly poor raw materials, such as wastes from the agro-food industry, sludge, etc. [3,11]. In the case of the latter, the solubilization of phosphorus compounds occurs after the application of the fertilizer to the soil environment. Also, phosphate rock is used as an addition to poor products [1,12,13,14]. Such phosphorus biofertilizers are enriched with sulfur, which is transformed into sulfuric acid by *Thiobacillus*.

Microorganisms may be considered a source of solubilizing agents for insoluble phosphates present in the form of hydroxyapatite (poultry bones and fish bones as well as phosphorite). *Acidithiobacillus ferrooxidans* (*A. ferrooxidans*) is one of the most common mesophilic and acidophilic ferrous iron and sulphur oxidizing bacterial strains [15], and one of the most important autotrophic bacterium involved in bioleaching of low-grade sulfide minerals. It has been used to dissolve non-metallic elements, for example, to recover arsenic from medicinal realgar [16,17,18,19], but also to solubilize phosphorus from rock phosphates. *A. ferrooxidans* is of special interest as a phosphate-solubilizing microorganism because of its natural ability to produce sulfuric acid, which can be used in the solubilization of hydroxyapatite [20]. Pyrites (FeS_2_) can be oxidized by *A. ferrooxidans*, producing H_2_SO_4_ and FeSO_4_. Rock phosphate is dissolved by H_2_SO_4_, forming soluble phosphorus. Fe^2+^ in FeSO_4_ is oxidized to Fe^3+^, producing energy to sustain the growth of *A. ferrooxidans* [21]. Yet its ability to oxidize ferrous ion and/or sulfur and at the same time to release phosphates has been found to be repressed to different degrees by the addition of naturally-occurring organic compounds in growth media [22].

Although rock phosphate can be successfully solubilized by these bacteria [23], the low release of soluble phosphate is a major challenge, since rock phosphate is poor in organic matter that enhances bacteria growth and at the same time induces a higher efficiency of solubilization. As far as we know, the release of soluble phosphate from phosphorus-bearing materials such as poultry bones and fish bones by *A. ferrooxidans* has never been studied, which is precisely why we cultivated *A. ferrooxidans* on the medium containing renewable materials in order to find out how to make its use in agriculture more effective and economical. To this purpose, we used poultry bones and fish bones as a raw material, which are a source of phosphorus and organic compounds that could increase the effectiveness of *A. ferrooxidans*-induced solubilization. Our objective was to investigate the possibility of phosphorus solubilization from poultry bones and fish bones, as well as phosphate rock apatite, using sulfuric acid generated by *A. ferrooxidans*.

## 2. Results and Discussion

Autotrophic acidophilic bacteria have a key role in the production of sulfuric acid, which critically affects the solubilization of phosphates. Sulfuric acid creates an acidic environment, and thus benefits the solubilization of the hydroxyapatite structure present in the poultry bones, fish bones and phosphate rock (phosphorite). Equation (1) presents the solubilization of hydroxyapatite:

Ca_5_(OH)(PO_4_)_3_ + 5H_2_SO_4_ + 10H_2_O → 5CaSO_4_·2H_2_O + 3H_3_PO_4_ + H_2_O (1)

whereas Equation (2) presents the solubilization of fluoroapatite present in phosphorite:

Ca_5_F(PO_4_)_3_ + 5H_2_SO_4_ + 10H_2_O → 5CaSO_4_·2H_2_O + 3H_3_PO_4_ + HF (2)

### 2.1. Changes in pH

The pH drops significantly during solubilization in most studied microorganisms, since most of them solubilize by the production of organic acids [24]. For *Bacillus megaterium*, the initial pH is close to neutral, so even though the pH drops to ca. 3.5, the ΔpH is much higher than that recorded for the solubilization performed by *A. ferrooxidans*, despite the fact that the final pH is much lower (since the starting point is lower).

An increase in pH was observed only in the case of phosphorite (10 g/L), as a result of the consumption of acid by the proton attack on rock phosphate, which was also reported by Xiao et al. [18]. Table 1 shows the comparison of initial and final as well as ΔpH, recorded for different doses of raw materials. The highest change in pH in the cultures was observed for the highest concentration of fish bones 20 g/L ΔpH = 0.861, while the lower changes in the pH were found in the cultures with higher doses of poultry bones (20 g/L). The value of the final pH increases as the poultry bone doses increase, while in the case of fish bones the value of the final pH decreases as the fish bone doses increase. The addition of fish bones could improve the pH reducing ability of autotrophic acidophilic bacterium *A. ferrooxidans*. This relationship between higher doses of fish bones and higher changes of pH may be explained by the fact that the metabolites of bacterial cells could interact and thus release various compounds from the fish bones, which could enhance the production of bacterial acid [25,26]. The relationship between the dose of poultry bones and the effect of the corresponding pH is consistent with observations presented previously [27]. Another explanation for the different pH effect for poultry bones and fish bones might be the chemical activity and stability that, in the case of fish bones, is lower because it represents the “younger” form of hydroxyapatite, when compared with poultry bones.

The utilization of hydroxyapatite of biological origin instead of phosphate rock had a significant influence on bacterial performance. The pH of the broth inoculated with the culture of *A. ferrooxidans* and with the addition of a renewable source of phosphorus was lower than that measured for solubilization with the use of rock phosphate. The addition of renewable resources can, on top of delivering phosphorus, deliver other compounds that potentially enhance the growth of bacterial cells and at the same time improve the pH-reducing ability of *A. ferrooxidans* by the production of higher amounts of acid or the synthesis of other organic molecules, which can affect the phosphates solubilization in other ways due to acid attacks on the fluorapatite structure [28].

### 2.2. Concentration of Phosphorus (Expressed as P_2_O_5_)

The changes in phosphorus concentrations during the solubilization experiments (expressed as P_2_O_5_) are described by the model $C_{P_{2}O_{5}}=f\left(t\right)$ presented below (Equation (3)).

(3) $$C_{P_{2}O_{5}}=C_{P_{2}O_{5}}^{max}\frac{K\cdot t}{1+K\cdot t}$$

where the $C_{P_{2}O_{5}}^{max}$, mg/L is the maximum concentration of phosphorus (expressed as P_2_O_5_) and K, 1/day is the constant that reflects the variable slope, which is called the Hill slope. When K is greater, the curve changes more sharply, and it means that the solubilization is performed faster.

**Figure 1:**
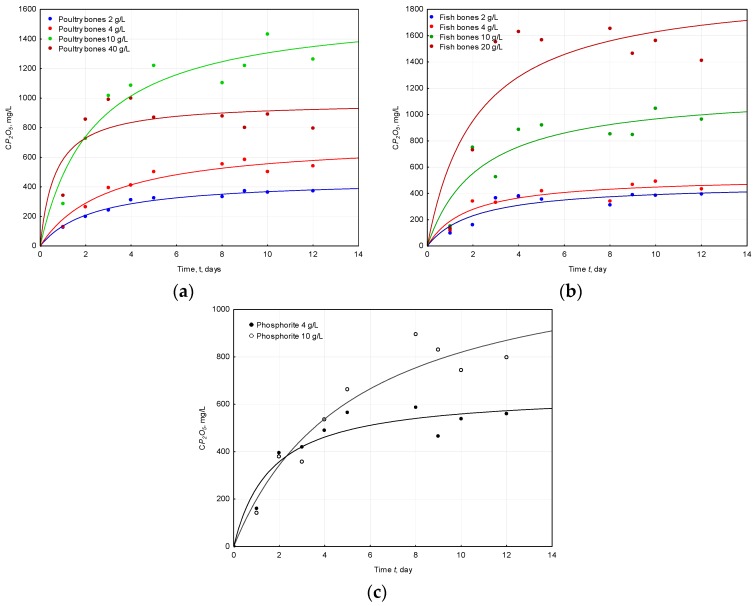
Changes of phosphorus (expressed as P_2_O_5_) concentrations during the solubilization performed by *A. ferrooxidans* with the utilization of three different phosphorus raw materials (**a**) poultry bones; (**b**) fish bones; and (**c**) phosphorite.

There was no influence of the doses of applied raw materials on the *K* (1/day). Only in the case of phosphorite higher doses of substrate raised the value of *K*, but in the case of the other parameters of the proposed model, there was a close relation between the maximum concentrations of phosphorus (expressed as P_2_O_5_) and the doses. The evaluated maximum concentration of P_2_O_5_ from the proposed model ranged between 454 and 1980 mg/L, with variations among different sources of phosphate and their doses (Table 2). The highest maximum concentration of 1.98% phosphorus (expressed as P_2_O_5_) was found for the solubilization performed on the highest dose of fish bones (20 g/L). The solubilization of apatite from poultry bones also resulted in high levels of 1.6% phosphorus (expressed as P_2_O_5_) obtained for the 10 g/L. An increase in the dose of poultry bones above 10 g/L results in lowering the $C_{P_{2}O_{5}}^{max}$ (Figure 1 and Table 2). This is probably because too high doses of poultry bones, or to be more precise, some organic or inorganic compounds originating from poultry bones, act as an inhibitor of solubilization [19].

The differences in the obtained concentrations of phosphorus (expressed as P_2_O_5_) between the same doses for different raw materials were found to be statistically significant: for a dose of 4 g/L the differences were: fish bones vs. phosphorite (*p* = 0.0288, *N* = 9); for 10 g/L: fish bones vs. phosphorite (*p* = 0.00828), poultry bones vs. phosphorite (*p* = 0.000246, *N* = 9), poultry bones vs. fish bones (*p* = 0.0865, *N* = 9); in the case of 20 g/L: poultry bones vs. fish bones (*p* = 0.0209). The observed differences might be a result of the components that were delivered with phosphorus by the different raw materials used in the experiment. In the case of bones (poultry and fish), many organic compounds such as amino acids, fatty acids, and lipids as well as minerals are delivered [29]. Lower concentrations of phosphorus (expressed as P_2_O_5_) were obtained for Morocco phosphorite when compared with the results obtained for bones. This can be explained by the origin of the phosphate rock. The immediate source of sedimentary phosphate is considered to be released from organic matter, which is degraded by microbially-mediated redox reactions [30]. Phosphate rock may occur with small amounts of organic matter that did not undergo phosphogenesis. These findings are consistent with the results described in the literature [24], where the growth rate of *B. megaterium* was monitored with the utilization of four different components of growth medium: poultry bones, fish bones, Morocco phosphorite and ash originating from the incineration of sludge from a waste water treatment plant. It was reported that phosphorite, when compared with ashes that are deprived of organic matter during incineration, served as a better source of nutrients since the plant growth rate was higher with the growth medium of phosphorite when compared with ashes which deliver only nutrients in an inorganic form. This, in turn, might have an effect on the affectivity of the solubilization of *A. ferrooxidans*. Additionally, some elements that may be released during organism growth in different compositions of the medium could affect the composition of the produced metabolites and, consequently, the microbial solubilization [29,31,32].

Another issue that may be the reason for the differences between the amounts of released phosphorus from bones and phosphorite by solubilizing bacteria is the fact that the chemical activity of the materials that have been used in the experiments varies significantly—a shorter formation time and a lower mineralization degree—bones—results in a higher solubilization efficiency; longer formation time and a higher mineralization degree—phosphorite—results in a lower solubilization efficiency [25].

### 2.3. Solubilization of Apatite

The solubilization effect or the Solubilization Factor (*SF*, %) is defined as the ratio (in percentage terms) of soluble P_2_O_5_ present in the solution and phosphorus (expressed as P_2_O_5_) introduced to the solubilization medium in solid form.

**Figure 2:**
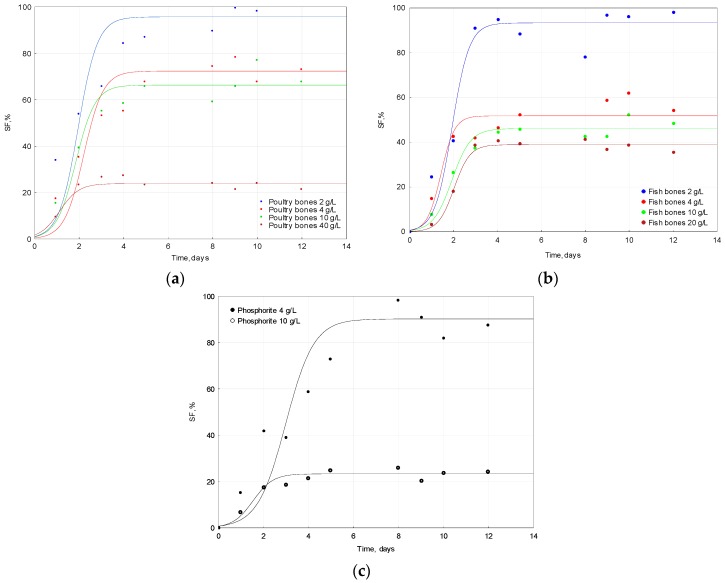
Solubilization Factor changes of phosphates in the process performed by *A. ferrooxidans* with the utilization of three different sources of phosphorus: (**a**) poultry bones; (**b**) fish bones; and (**c**) phosphorite.

Attempts to describe the changes of the Solubilization Factor in time were undertaken with the use of the model (Equation (4)):

(4) $$SF=f\left(t\right)=\frac{SF_{max}}{1+10^{k\left(L-x\right)}}$$

where *SF_max_*, % is the maximum solubilization factor, *L* is the time at which the *SF* is equal to ½ of *SF_max_* and *k*, 1/day constant is the variable slope which is called the Hill slope. When *k* is greater, the curve changes more sharply, and it means that solubilization occurs faster. Figure 2 shows the changes of the phosphates solubilization factor in the process performed with the utilization of phosphorus resources. Table 2 collects all parameters of the proposed model together with the *p*-value and errors that express the fit of the model to the experimental data.

The evaluated parameter *SF_max_* for bones was found to be statistically significant and correlated with the raw materials doses applied in the experiment, when the relationship was described as a linear regression: f(dose) = *SF*: *y* = 96.8 − 3.58*x*, R^2^ = 0.937; *p* = 0.032. Another empirical model was used to describe the experimental points in the case of fish bones solubilization, since the linear regression did not describe the experimental data well. The following model was used: *SF* % = (A + (B* Dose of raw material))/Dose of raw material [27]. It yielded the following parameters: A = 118 mg/L (*p* = 0.0292), a constant describing the reaching plateau, and B = 30.9% (*p* = 0.0346) that can be interpreted as the minimal value of *SF* %. The proposed model gave a better fit (R^2^ = 0.975, χ^2^ = 1.79) to the experimental points than the linear regression (R^2^ = 0.544; *p* = 0.262).

Parameter k, which reflects the solubilization rate, has been found to increase when the dose of the substrate increases. For poultry bones, this relation was statistically significant: f(dose) = *k*: y = 0.129 − 0.0749x, R^2^ = 0.917; *p* = 0.042 (Table 2). For fish bones, the linear regression did not result in a high correlation coefficient (R^2^ = 0.675; *p* = 0.178).

The doses of phosphorus substrates did not strongly affect parameter L in all discussed cases, but small differences occurred between the average of parameter L and used materials; 1.78 ± 0.468; 1.83 ± 0.272 and 2.24 ± 1.02 for poultry bones, fish bones and phosphate rock, respectively.

As it was discussed above, two empirical models were used to describe the changes in *SF* and phosphorus concentrations (expressed as P_2_O_5_) over time. Table 3 summarizes the relationship between the parameters of both models. It was found that *K*, 1/day (($f\left(t\right)=C_{P_{2}O_{5}}$)) and *k*, 1/day (f(t) = *SF*) were statistically correlated (r = 0.794, *p* < 0.05), and since both describe the performance of solubilization, this correlation is consistent with the model assumptions. A strong correlation was also discovered between *K* 1/day (($f\left(t\right)=C_{P_{2}O_{5}}$)) and *L*, day (f(t) = *SF*) (r = 0.7978, *p* < 0.05).

The high dose of the substrate had a negative influence on the biosolubilization of phosphates. This phenomenon was observed in the case of poultry bones and fish bones, as well as phosphorite. These findings are consistent with the results reported in the literature [24,25,27]. The highest solubilization factor was reached for the lowest doses of substrates: 95.7% for 2 g/L of poultry bones, 93.0% for 2 g/L of fish bones and 90.8% for 4 g/L of phosphorite. Similar results were reported in the literature [8].

The solubilization factor has previously been reported to be much higher when compared with the solubilization performed by *B. megaterium* [25,27], probably because acids produced by *B. megaterium* are much weaker than the sulfuric acid produced by *A ferrooxidans*. The drop in pH is lower for solubilization performed by *A. ferrooxidans* than that by *B. megaterium*. When the dose of the substrate used in the experiments was doubled, the *SF* decreased by 24%, 45% and 74% for poultry bones, fish bones and phosphorite, respectively.

The fraction of phosphorus solubilized from rock phosphate by biosolubilization performed by *A. ferrooxidans* was reported by Chi et al. to be up to 11.8% [33]. The reason for such significantly higher solubilization effectiveness in the case of phosphorite (up to 91%) presented in this paper, might be the growth medium composition, where FeSO_4_ was used instead of pyrite FeS_2_, as reported by Chi et al. [33]. Table 3 presents a matrix correlation of the concentrations of elements that occur in the microbial broth after the solubilization in all discussed cases. A statistically significant correlation was found between P and Ca (r = 0.209, *p* = 0.048): it is two times lower than the molar ratio in the hydroxyapatite (0.465). Many statistically significant correlations were observed between sulfur and others elements, such as Fe (r = 0.818), K (r = −0.587), Mg (r = −0.587), Mn (r = −0.566), Na (r = −0.0531) and Ni (r = 0.516), which is consistent with the solubilization mechanism performed by *A. ferrooxidans*, since sulfuric acid is produced and affects the release of many compounds present in the raw materials used as a phosphorus source.

The phosphate rock, as well as other materials used in this study, meet the necessary requirements for biofertilizers, primarily because of the relatively low content of P_2_O_5_ (0.08% of P_2_O_5_ in the case of the fertilizer obtained from poultry bones—20 g/L; 0.14% of P_2_O_5_ in the case of the fertilizer obtained from fish bones—20 g/L), but also because of the low content of toxic elements (Cd, Cu, Cr, Ni, Pb, Zn). Biogenic apatite (bone) has the lowest total concentration of all elements. The low concentration of impurities in biogenic apatite is the result of a short accumulation time (bone formation takes a few weeks) [34]. Another advantage of the application of bones is the lack of fluorine, which plays a major role in sedimentary apatite formation and contributes to its preservation in sediments [35,36]. Multi-elemental analysis (ICP-OES) (Table 4) of the content of toxic elements and heavy metals in the formulations based on renewable raw materials was lower as compared to their presence in the formulation obtained with the utilization of phosphorite, with one exception of Ni that, in the case of fish bones, was 50% higher when compared with phosphorite. All findings are below the acceptable levels of toxic elements, which is essential for potential biofertilizers. Literature data demonstrated that fish bones show a higher quality than poultry bones in terms of chemical impurities (i.e., magnesium and alkali metals), which is an added advantage of fish bones utilization over poultry bones utilization.

## 3. Materials and Methods

### 3.1. Microorganisms

Solubilizing bacteria *A. ferrooxidans* used in the experiments were obtained from Professor Zygmunt Sadowski from Wroclaw University of Technology. *A. ferrooxidans* is an autochthonous strain of bacteria (F7-01) isolated from the tailings impoundment “Iron Bridge,” Poland.

**Figure 3:**
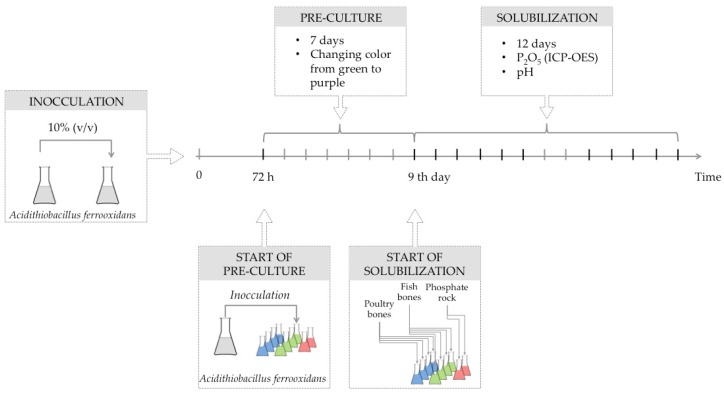
The scheme sampling timing of the experiment.

The strain was cultivated in 9 K medium of Silverman and Lundgren [37] composed of 3 g (NH_4_)_2_SO_4_, 0.5 g MgSO_4_·7H_2_O, 0.5 g K_2_HPO_4_, 0.1 g KCl, 0.01 g Ca(NO_3_)_2_ and 44.2 g FeSO_4_·7 H_2_O (per liter of distilled water), containing elemental sulfur (0.8 g/L). The pH of the medium was adjusted to 2.5 with H_2_SO_4_ [32] prepared with technical grade reagents (from POCh S.A., Gliwice, Poland). The inoculation was undertaken by transferring 10% (*v*/*v*) of 72 h-old well-grown culture of *A. ferrooxidans* (cell density—1.0 × 10^8^ CFU·mL^−1^) to fresh medium (Figure 3). During the experiment’s first seven days, pre-cultures were conducted at 30 °C without the addition of phosphorus-bearing substrates to enable the bacteria to utilize acids present in the solution and ensure sufficient time for bacterial cells to produce their own sulfuric acid. The color of the suspension changed from green to purple since, at the growth stage, bacteria oxidize iron ions (from Fe^2+^ to Fe^3+^). After the initial cultivation of bacteria, the given amounts (mentioned below in Section 3.2) of phosphorus-bearing materials were added to Erlenmeyer’s flasks where the initial phase was undertaken and the main solubilization experiment was conducted.

### 3.2. Solubilization Experiments

Solubilization experiments were conducted for three different types of phosphorus raw material: crushed boiled poultry bones (18.6% P_2_O_5_) [25], crushed fish bone (20.0% P_2_O_5_), and ground Morocco phosphorite (22.7% P_2_O_5_) [25]. All samples were pulverized and sieved to pass through 1 mm particle size fractions for chemical and solubilization studies.

Four doses of poultry bones and fish bones were used in the experiment (2, 4, 10 and 20 g/L), and two doses (2 and 4 g/L) were used in the case of phosphorite. Ten Erlenmeyer’s flasks, containing 250 mL of medium for *A. ferrooxidans* (four with medium containing poultry bones and four with medium containing fish bones as well as two with phosphorite as a phosphorus source) were used in the experiment. All experimental samples were carried out in triplicate. All presented values are an arithmetic mean.

The solutions were sterilized and then inoculated with *A. ferrooxidans*. During 12 days of cultivation/solubilization (Figure 3), culture media were shaken and incubated at 34 °C (Thermoshake Gerhardt, Bonn, Germany). The process was performed in the batch culture mode. Samples of microorganism suspension from both culture groups were collected at the same time. The permeates were used for the evaluation of pH and P_2_O_5_ concentrations that were measured by the ICP-OES method [38]. The pH measurements were conducted with the Mettler-Toledo (Seven Multi, Greifensee, Switzerland) pH-meter equipped with an electrode InLab413 compensating for temperature.

### 3.3. Analytical Methods

One half milliliter of permeates were digested with 2.5 mL concentrated—65% *m*/*m* HNO_3_ suprapur grade from Merck (Darmstadt, Germany) in Teflon vessels (microwave oven Milestone MLS-1200). After mineralization, all samples were diluted to 50 mL. The concentrations of elements in all digested and diluted samples were determined by means of the Inductively Coupled Plasma-Optical Emission Spectrometer (Varian VISTA-MPX ICP-OES, Victoria, Australia) fitted with an ultrasonic nebulizer (U5000AT+, CETAC, Omaha, UNO, USA) in the Chemical Laboratory of the Multielemental Analyses at Wroclaw University of Technology, which is accredited by ILAC-MRA and the Polish Centre for Accreditation according to PN-EN ISO/IEC 17025 (nr AB 696) [38].

### 3.4. Calculations

The arithmetic mean values, standard deviations (SD) and t tests as well as the model parameters of equations describing the experimental data were determined with nonlinear estimation and multiple regression modules of Statistica software ver. 9.0 (StatSoft, Krakow, Poland). Correlation was considered statistically significant at *p* < 0.05.

Chi-square test (χ^2^ test) was also used, which was calculated from Equation (5), which more accurately described the fit of the model to the experimental data compared to the determination coefficient R^2^.

(5) $$\chi^{2}=\frac{{\left(experimental\;value-model\;value\right)}^{2}}{model\;value}$$

## 4. Conclusions

To become available for plants, phosphorus needs to be converted into a soluble form; this solubilization occurs in nature in the presence of a large number of acidogenic autotrophs and heterotrophs (bacteria, fungi and yeasts), which produce inorganic or organic acids with which they are capable of dissolving insoluble phosphates.

The results presented here demonstrate the usefulness of *A. ferrooxidans* in the biosolubilization of phosphorus occurring in poultry bones, fish bones and phosphorite. The solubilization factor was the highest for the lower dose of poultry bones (96%) and fish bones (94%), as well as for phosphorite (91%). Fertilizers obtained from the solubilization performed with the highest doses of phosphorus substrates contained 1.6% and 1.9% of phosphorus (expressed as P_2_O_5_) for poultry bones and fish bones, respectively. The formulations can be classified as organic phosphorus fertilizers in liquid form, pursuant to the Regulation of the Minister of Agriculture and Rural Development of Poland on the Implementation of Certain Provisions of the Act on Fertilizers and Fertilization (Journal of Laws of 2004, no. 236, item. 2369 &14), which specifies that the minimum quality requirements for the phosphorus content (expressed as P_2_O_5_) in phosphorus fertilizers is 0.05% (m/m).

## Figures and Tables

**Table 1:** The comparison of initial and final as well as ΔpH recorded for different doses of raw materials.

Raw Material	Dose	pH Initial	pH Final	ΔpH
POULTRY BONES	2	2.495	1.65	0.845
	4	2.529	1.744	0.785
	10	2.484	1.832	0.652
	20	2.518	1.895	0.623
FISH BONES	2	2.51	1.753	0.757
	4	2.489	1.7	0.789
	10	2.497	1.643	0.854
	20	2.498	1.637	0.861
PHOSPHATE ROCK	4	2.494	1.853	0.641
	10	2.528	2.720	−0.192

**Table 2:** Parameters of the model describing P_2_O_5_ concentration changes during solubilization $C_{P_{2}O_{5}}=f\left(t\right)=C_{P_{2}O_{5}}^{max}\frac{K\cdot t}{1+K\cdot t}$ (left side), and those describing the Solubilization Factor change during solubilization $SF=f\left(t\right)=\frac{SF_{max}}{1+10^{k\left(L-x\right)}}$ (right side). SE—standard error; RM—raw material; *p*—*p*-value.

RM	Dose g/L	$C_{P_{2}O_{5}}=f\left(t\right)=C_{P_{2}O_{5}}^{max}\frac{K\cdot t}{1+K\cdot t}$						$SF=f\left(t\right)=\frac{SF_{max}}{1+10^{k\left(L-x\right)}}$					
		Parameter	Value	SE	*p*	R^2^	χ^2^	Parameter	Value	SE	*p*	R^2^	χ^2^
POULTRY BONES	2	*C_max_P_2_O_5_*, mg/L	454	18.1	0.000	0.993	6.17	*SF_max_*, %	95.7	3.4	0.000	0.982	16.1
		*K*, 1/day	0.424	0.056	0.000			*k*, 1/day	0.418	0.078	0.001		
								*L*, day	1.95	0.21	0.000		
	4	*C_max_P_2_O_5_*, mg/L	722	58	0.000	0.981	36.54	*SF_max_*, %	72.4	2.5	0.000	0.985	9.30
		*K*, 1/day	0.335	0.0805	0.00315			*k*, 1/day	0.435	0.077	0.001		
								*L*, day	2.21	0.20	0.000		
	10	*C_max_P_2_O_5_*, mg/L	1614	148	0.000	0.968	150.5	*SF_max_*, %	66.3	2.4	0.000	0.980	7.49
		*K*, 1/day	0.424	0.129	0.0111			*k*, 1/day	0.619	0.146	0.004		
								*L*, day	1.85	0.19	0.000		
	20	*C_max_P_2_O_5_*, mg/L	975	98	0.000	0.904	231	*SF_max_*, %	23.9	0.8	0.000	0.978	1.47
		*K*, 1/day	1.49	0.98	0.164			*k*, 1/day	1.74	1.32	0.229		
		*C_max_P_2_O_5_*, mg/L						*L*, day	1.12	0.12	0.000		
FISH BONES	2	*K*, 1/day	474	63	0.000	0.931	95.1	*SF_max_*, %	93.9	4.0	0.000	0.972	15.9
		*C_max_P_2_O_5_*, mg/L	0.474	0.220	0.0632			*k*, 1/day	0.784	0.248	0.016		
		*K*, 1/day						*L*, day	1.94	0.20	0.000		
	4	*C_max_P_2_O_5_*, mg/L	529	53	0.000	0.950	62.3	*SF_max_*, %	51.8	2.8	0.000	0.949	9.18
		*K*, 1/day	0.560	0.213	0.0304			*k*, 1/day	0.891	0.373	0.048		
		*C_max_P_2_O_5_*, mg/L						*L*, day	1.42	0.25	0.001		
	10	*K*, 1/day	1189	169	0.000	0.929	286	*SF_max_*, %	46.1	1.4	0.000	0.961	3.61
		*C_max_P_2_O_5_*, mg/L	0.435	0.205	0.0664			*k*, 1/day	0.716	0.155	0.002		
		*K*, 1/day						*L*, day	1.92	0.15	0.000		
	20	*C_max_P_2_O_5_*, mg/L	1980	342	0.000	0.898	763	*SF_max_*, %	38.9	0.9	0.000	0.992	3.36
		*K*, 1/day	0.464	0.277	0.132			*k*, 1/day	1.403	0.492	0.025		
		*C_max_P_2_O_5_*, mg/L						*L*, day	2.02	0.07	0.000		
PHOSPHATE ROCK	4	*K*, 1/day	1256	187	0.000	0.971	86.3	*SF_max_*, %	90.8	4.5	0.000	0.971	7.29
		*C_max_P_2_O_5_*, mg/L	0.188	0.0637	0.0186			*k*, 1/day	0.292	0.072	0.007		
		*K*, 1/day						*L*, day	2.96	0.33	0.000		
	10	*C_max_P_2_O_5_*, mg/L	651	55	0.000	0.961	65.3	*SF_max_*, %	23.7	1.0	0.000	0.945	1.45
		*K*, 1/day	0.607	0.203	0.0171			*k*, 1/day	0.532	0.170	0.020		
								*L*, day	1.52	0.24	0.001		

**Table 3:** The table of correlation factors between the parameters of considered kinetic describing models (* *p* < 0.1; ** *p* < 0.05), *N* = 10.

	$\begin{array}{l} C_{P_{2}O_{5}}=f\left(t\right)\\ =C_{P_{2}O_{5}}^{max}\frac{K\cdot t}{1+K\cdot t} \end{array}$		$SF=f\left(t\right)=\frac{SF_{max}}{1+10^{k\left(L-x\right)}}$		
	$C_{P_{2}O_{5}}^{max}$	*K*	*k*	*SF*	*L*
$C_{P_{2}O_{5}}^{max}$	*1.00*				
***K***	−*0.095*	1.00			
***K***	*0.314*	0.794 **	1.00		
***SF***	−*0.269*	−0.618 *	−0.628 *	1.00	
***L***	*0.261*	−0.778 **	−0.618 *	0.665 **	1.00

**Table 4:** The content of toxic elements present in the obtained formations (mg/kg) (*N* = 10), mean ± SD, and acceptable limits of their content in the fertilizers pursuant to the Regulation of the Minister of Agriculture and Rural Development of Poland on the Implementation of Certain Provisions of the Act on Fertilizers and Fertilization (Journal of Laws of 2004 No. 236, item 2369). a–j indicate statistically significant differences at *p* < 0.05.

Formulations	Cd	Cu	Cr	Ni	Pb	Zn
**Poultry Bone**	0.0874 ^a^ ± 0.1095	0.239 ^b^ ± 0.473	0.450 ^c^ ± 1.991	3.81 ^k^ ± 1.26	2.67 ± 2.16	2.58 ^d^ ± 2.33
**Fish Bone**	0.104 ^e^ ± 0.089	0.253 ^f^ ± 0.221	0.0214 ^g^ ± 0.0506	5.05 ^h,k^ ± 2.06	3.04 ± 2.14	3.51 ^j^ ± 2.29
**Phosphorite**	0.260 ^a,e^ ± 0.167	0.671 ^b,f^ ± 1.087	2.66 ^c,g^ ± 5.12	3.54 ^h^ ± 1.46	3.39 ± 3.75	7.36 ^d,j^ ± 4.24
**Acceptable limits**	3 mg/kg	400 mg/kg	100 mg/kg	30 mg/kg	100 mg/kg	1500 mg/kg
